# Better Living through Chemistry: Caloric Restriction (CR) and CR Mimetics Alter Genome Function to Promote Increased Health and Lifespan

**DOI:** 10.3389/fgene.2016.00142

**Published:** 2016-08-18

**Authors:** Zoe E. Gillespie, Joshua Pickering, Christopher H. Eskiw

**Affiliations:** ^1^Department of Food and Bioproduct Sciences, University of SaskatchewanSaskatoon, SK, Canada; ^2^Department of Biochemistry, University of SaskatchewanSaskatoon, SK, Canada

**Keywords:** caloric restriction, caloric restriction mimetics, gene expression, epigenetics/genetics, aging, cancer

## Abstract

Caloric restriction (CR), defined as decreased nutrient intake without causing malnutrition, has been documented to increase both health and lifespan across numerous organisms, including humans. Many drugs and other compounds naturally occurring in our diet (nutraceuticals) have been postulated to act as mimetics of caloric restriction, leading to a wave of research investigating the efficacy of these compounds in preventing age-related diseases and promoting healthier, longer lifespans. Although well studied at the biochemical level, there are still many unanswered questions about how CR and CR mimetics impact genome function and structure. Here we discuss how genome function and structure are influenced by CR and potential CR mimetics, including changes in gene expression profiles and epigenetic modifications and their potential to identify the genetic fountain of youth.

## Introduction

The aging process is undoubtedly the single most significant contributor to disease and death. Although this has been the inevitable outcome of all life on this planet, is aging an unavoidable consequence or can it be treated and potentially cured? As of yet this question remains unanswered, but many believe that the aging process is essentially a disease. Environmental conditions, including lifestyle, can greatly affect the rate of aging. For example, obesity or excessive ingestion of calories has been linked to increased incidents of age-related pathologies such as diabetes, cardiovascular disease, stroke, type II diabetes, and cancer development (Fontana and Klein, 2007). Several lines of research indicate that certain behaviors can increase our health and potentially lifespan, such as exercise and regimes to improve cardiovascular function. One such intervention is the use of dietary/caloric restriction (CR); the reduced intake of calories/nutrients without causing malnutrition. This was first recognized over 80 years ago in dietary restricted rats exhibiting increased mean and maximal lifespans (McCay et al., 1935). In recent years, this observation has been verified across a large number of model organisms (Longo, 2009; Lee and Longo, 2016). These observations not only demonstrated an increase in the lifespan, but also in healthspan (time spent being healthy) of these organisms coincident with a significant decrease in age-related pathologies such as cardiovascular disease, diabetes and a number of cancers. For example, when fed a diet consisting of 35% of the *ad libitum* intake but enriched with vitamins and minerals, mice lived an average of 53 months, compared to 35 months in the control *ad libitum*-fed group (Weindruch et al., 1986). In addition, preliminary research indicates that CR prior to chemotherapy in humans increases tumor responsiveness while reducing side effects (Lee and Longo, 2011). Several drugs or naturally occurring compounds in food (nutraceuticals) have been found to “mimic” the phenotypes of CR (Lee and Min, 2013) and could be potential alternatives to this somewhat difficult to follow dietary regime. An obvious first question: Do these compounds mirror the effects of CR? A large body of outstanding research focuses on the impact of CR and mimetics on autophagy (*self-eating*) in the regulation of longevity and in promoting apoptosis in cancer cells [from groups such as Drs. S. Pattingre and G. Kroemer (for an extensive review see Klionsky et al., 2008)]; however, the mechanism and impact on genome function (gene expression) and organization (epigenetic changes and physical genome folding) are less well understood. In this review, we will discuss the effect of CR on genome function and structure and compare the impact of CR mimetics in an attempt to gage their ability to increase health and lifespan at the genetic level.

## Potential impact of CR on how we age

**Figure 1:**
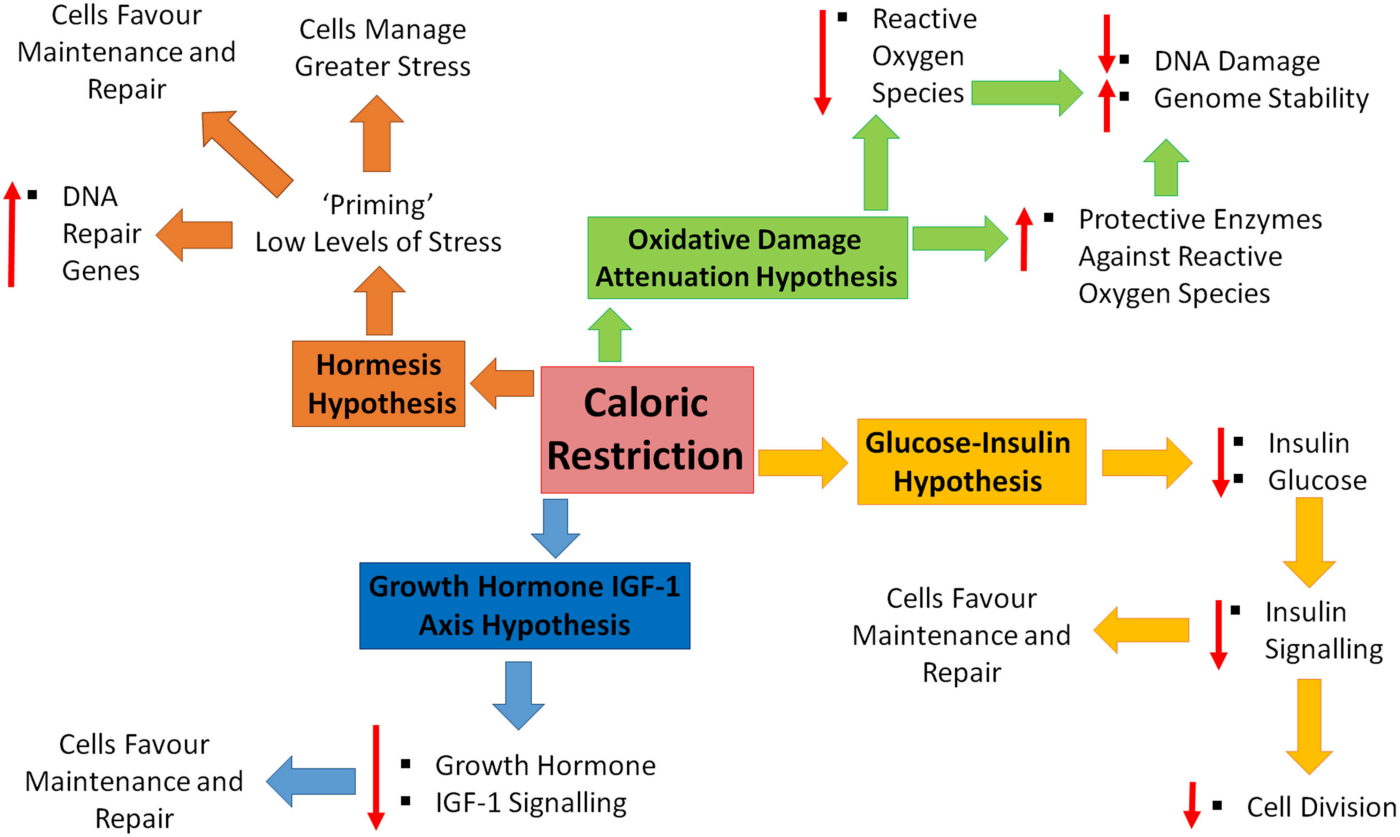
**Caloric restriction and the four hypotheses of aging**. Four major theories of aging; oxidative damage attenuation hypothesis (green), glucose-insulin hypothesis (yellow), growth hormone and insulin-like growth factor (IGF)-1 (blue), and the hormesis hypothesis (orange). Oxidative damage is decreased during caloric restriction (CR), through decreased production of reactive oxygen species and the up-regulation of protective enzymes, resulting in a decrease in DNA damage and increase in genome stability. CR causes decreased levels of circulating insulin and glucose, resulting in decreased cell growth and division, shifting toward maintenance and repair. Decreased levels of growth hormone and IGF-1 in response to CR, promoting maintenance and repair. CR is inducing a low level of stress which enables cells to counteract higher stresses, increase DNA repair gene expression and favor maintenance and repair.

Four major theories have been proposed to explain how/why we age. These theories include; increased reactive oxygen species (ROS) and decreased DNA repair, increased circulating glucose and insulin, increased circulating growth hormone and insulin-like growth factor-1 and the hormesis hypothesis. CR has been shown to promote increased lifespan and slow the aging process (see Figure 1 for a brief summary). How does CR exhibit these effects and fit in within these proposed theories?

## CR reduces levels of reactive oxygen species and increases DNA repair

The oxidative damage attenuation hypothesis states that increased metabolism from high levels of nutrients/calories leads to higher rates of ROS and that lowering these levels will prevent lipid, protein and DNA damage (Ray et al., 2012). Damage such as this would lead to decreased function of cellular components as well as to increased rates of mutation. However, the other side of this hypothesis states that lower metabolic rates results in decreased rates of DNA damage and increased genome stability, and thus in fewer incidents of cancer. This in itself would promote increased lifespan through improved genomic “health.” Furthermore, CR resulted in a reversal of age-related DNA damage in multiple mouse tissues by inducing genes from the base excision repair (BER) pathway (Cabelofa et al., 2003) and by modifying the activity of the Ku70/80 proteins (Um et al., 2003). In addition, many of the premature aging diseases, such as Werner syndrome (Sidorova, 2008) and Hutchinson Gilford Progeria syndrome (HGPS; Cao et al., 2011; Musich and Zou, 2011), have increased levels of DNA damage and lower rates of DNA repair. The rapid accumulation of DNA damage likely drives these cells into premature senescence leading to rapid onset of aging-phenotypes. Although this is logical, some data does indicate that there is not a significant enough change in free radical production upon CR to significantly decrease ROS levels (Miwa et al., 2004) indicating that the benefits of CR might not be elicited through this mechanism.

Although CR increases lifespan, it may not be due to a reduction of the ROS levels produced by mitochondria (Miwa et al., 2004), but may result from an increase in the expression of enzymes that protect against these highly reactive molecules, reducing net oxidative stress. However, in *D. melanogaster* exposed to CR, no link between lifespan extension and increased resistance to oxidative stress has been found. Furthermore, CR of older flies significantly decreases resistance to oxidative stress with both cytosolic superoxide dismutase 1 (SOD1) and mitochondrial SOD2 unresponsive to CR, contrary to the proposal that CR causes life span extension by increasing resistance to oxidative stress (Kabil et al., 2007). These observations support the suggestion that CR may not elicit its beneficial properties via reduction in ROS or resistance or oxidative stress.

## CR decreases circulating glucose and insulin

The altered glucose-insulin hypothesis indicates that CR causes a decrease in the circulating levels of both insulin and glucose, leading to decreased insulin signaling. This is based on observations that decreased insulin signaling promotes increased lifespan in a variety of model organisms, including *C. elegans* (worms; Murphy and Hu, 2013), *D. melanogaster* (flies; Kannan and Fridell, 2013), and *M. musculus* (mouse; Zhang and Liu, 2014). Increased glucose and insulin in the circulatory system will cause peripheral cells to absorb this glucose and convert it to ATP. In addition, insulin will also send positive growth and proliferative signals, pushing cellular balance toward growth and cell division. Increased metabolism will produce ROS from mitochondria as well as shortening the time in which cells repair or replace old or damaged molecules. Although enough energy is present to produce daughter cells, these rapid rates of cell division may be harmful. Therefore, CR may promote increased lifespan by decreasing rates of cell division and favoring repair and maintenance.

## CR decreases circulating growth hormone (GH) and insulin-like growth factor-1 (IGF-1)

The growth hormone-IGF-1 axis hypothesis states that increased signaling through these pathways advances the aging process by promoting cell growth and proliferation. Similarly to the glucose-insulin level hypothesis, CR causes the reduction of growth hormone/IGF-1 signaling (Fontana et al., 2015), favoring a switch from cell growth and proliferation to maintenance and repair in mice. CR phenotypes parallel long lived Ames and Snell dwarf mice, with either decreased GH receptor activity or knock-outs of the growth hormone binding protein gene exhibiting hepatic synthesis of IGF-1, reduced secretion of insulin, increased hepatic sensitivity to insulin, reduced plasma glucose, and increased resistance to oxidative stress (Brown-Borg and Bartke, 2012). However, in human studies Fontana and colleagues documented that over a 2 year period of CR, no change in circulating IGF-1 levels were observed (Fontana et al., 2008, 2015). These findings hint at two potential conclusions; (1) CR does not work in humans, only in mice, or (2) CR does not impact IGF-1 levels; however, it does impact other pathways, leading to at least increased healthspan, if not lifespan.

## CR alters cell behavior from proliferation and growth to maintenance and repair

The hormesis hypothesis states that low levels or intensity of stress leads to “priming” in which cells/tissues/organs can then withstand other stresses that would normally prove terminal. It is thought that with hormesis, cells move from active growth and proliferation to a state that favors repair and maintenance. CR may prime cells by activating stress pathways to deal with later assault such as DNA damage. CR is documented to increase the expression of genes involved with DNA repair (Cabelofa et al., 2003) which will increase the efficiency at which cells are able to cope with, for example, oxidative damage. Other specific observations appear to favor this model, activating transcription factors and mechanisms controlling gene expression leading to increased levels of proteins mediating cellular stress responses (Calabrese et al., 2011).

## How are nutrients sensed at the cellular level?

**Figure 2:**
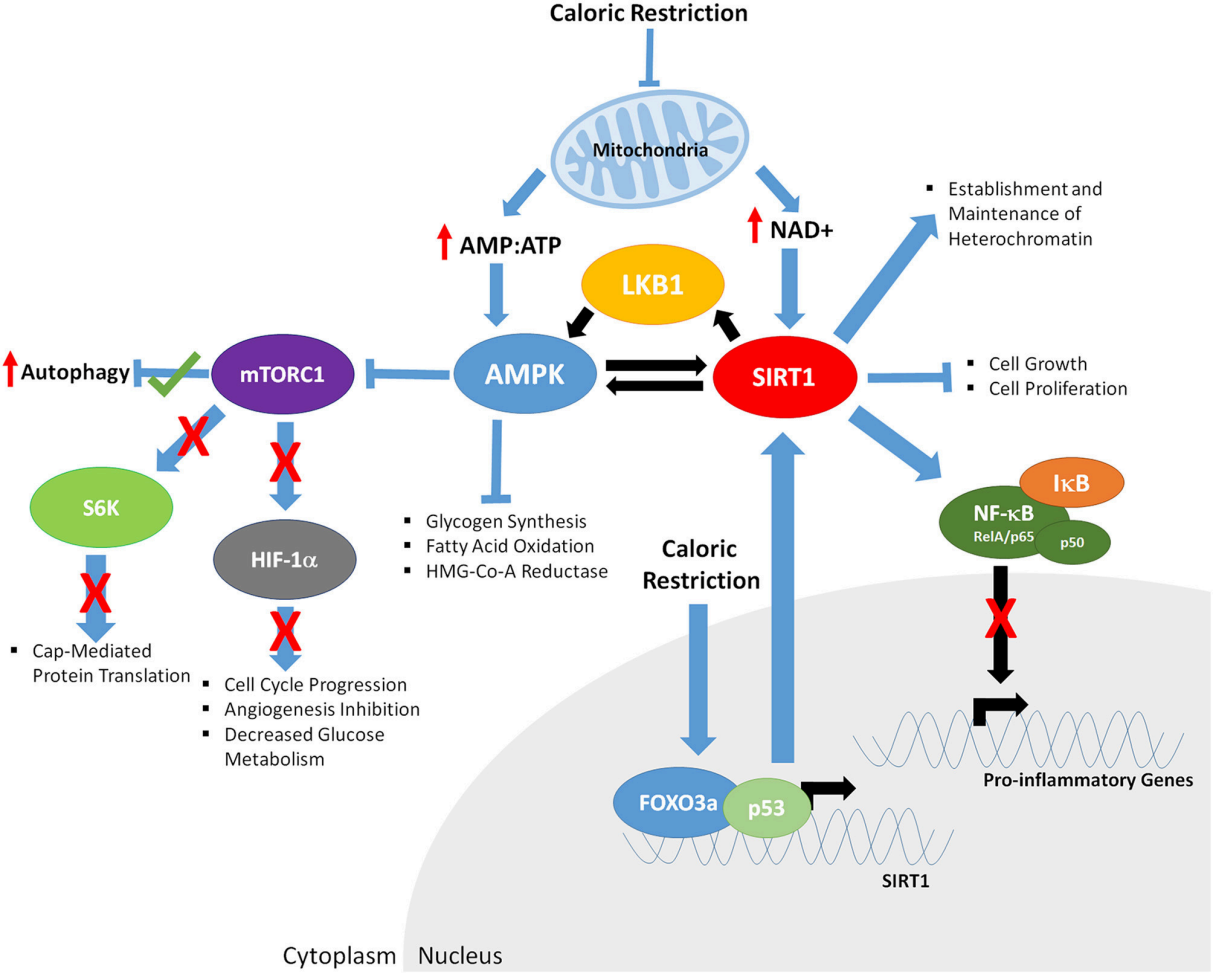
**The downstream effects of caloric restriction on key aging mediators AMPK and SIRT1**. CR results in decreased mitochondrial function, increasing the ratio of AMP:ATP and levels of NAD+. Increased AMP:ATP ratio leads to activation of AMPK. Furthermore, AMPK is phosphorylated/activated by LKB1 and SIRT1-mediated deacetylation, generating a feedback loop. AMPK inhibits glycogen synthesis, fatty acid oxidation, HMG-Co-A Reductase, and mTOR function (indirectly through phosphorylation of the TSC1/2 complex; not shown). Decreased mTORC1 function results in; increased autophagy, decreased S6K activity and cap mediated protein translation, inhibits HIF-1α, resulting in reduced cell cycle progression, angiogenesis and glucose metabolism. Increased NAD+ levels promote SIRT1 activity, resulting in deacetylation of LKB1 and AMPK, and upregulation of their respective function. CR enables binding of FOXO3a and p53 in the promoter of SIRT1, further driving SIRT1 expression. Activated SIRT1 deacetylates the RelA/p65 component of NFκB, preventing degradation of IκB and sequestering NF-κB in the cytoplasm, inhibiting proinflammatory gene expression. SIRT1 also promotes establishment and maintenance of heterochromatin and decreases cell growth and proliferation.

Although it is clear that CR promotes increased health and lifespan (although this is controversial in primates) several questions still remain unanswered. For example, do all cell types share common mechanisms mediating CR to promote increased cell longevity? Two major cellular energy sensors have been identified, the adenosine monophosphate kinase (AMPK) and the Sirtuin 1 (SIRT1) deacetylase (Figure 2). AMPK is activated as the ratio of cellular AMP:ATP rises, indicating low levels of energy. AMPK regulates both catabolic and anabolic processes in response to energy levels (Gowans and Hardie, 2014) and has a variety of targets. When active, AMPK inhibits glycogen synthesis, fatty acid oxidation and HMG-Co-A reductase (Lim C. T., et al., 2010). Further, it has been suggested that chronic activation of AMPK results in expression of muscle hexokinase and glucose transporters (Glut4), mimicking the effects of extensive exercise training (Zhou et al., 2001). AMPK further activates proteins involved with fatty acid hydrolysis, glucose up-take and p53 signaling. Downstream of AMPK is the target of rapamycin (TOR) signaling cascade. TOR is evolutionarily conserved and functions in mediating growth and environmental cues; therefore, it likely plays a central role in regulating cellular responses to CR and CR mimetics. Comprised of two individual complexes, TORC1 and TORC2 (Yang et al., 2013), TOR further mediates signals from a variety of upstream cascades, including the insulin (Bjedov et al., 2010; Verges and Cariou, 2015) and PI3K pathways (Massacesi et al., 2016). Low cellular energy levels promote AMPK to phosphorylate tuberous sclerosis complex (TSC)-1 and 2 which in turn inhibit TORC1. This decrease in TORC1 function leads to decreased S6 kinase phosphorylation, leading to decreased rates of cap-mediated protein translation. AMPK has also been shown to activate and repress specific transcription factors involved with modulating gene expression. AMPK also phosphorylates SIRT1, further leading to its activation. SIRT1 is stimulated in response to decreased energy levels, leading to an increase in cellular concentration of nicotinamide adenine dinucleotide (NAD+; Lau et al., 2014). SIRT1 deacetylates both histone and non-histone targets, in the nucleus and the cytoplasm, leading to changes in cellular function and gene expression. One of the targets of SIRT1 is the LKB1 kinase. When LKB1 is deacetylated, it phosphorylates AMPK, further driving AMPK activation. Both AMPK and SIRT1 have common targets, such as p53, implicating these proteins as central in controlling CR mediated changes in gene expression and cellular function, leading to increased health and longevity.

Parallel to AMPK, the General Control Nonderepressible (GCN2) factor detects decreased levels of amino acids through sensing of uncharged tRNAs. GCN2 decreases rates of translation, preserving limited pools of amino acids as well as conserving energy (Chantranupong et al., 2015). Furthermore, GCN2 activates the ATF4 transcription factor which then regulates the expression of genes involved with apoptosis, autophagy, and amino acid metabolism, including up-regulation of select amino acyl tRNA synthetases and amino acid transporters (Harding et al., 2000, 2003; Bunpo et al., 2010; B'Chir et al., 2013; Krokowski et al., 2013). Furthermore, Sestrin2 binds the pentameric GTPase activating TOR (GATOR) 2 complex under amino acid depleted conditions (in particular leucine), releasing GATOR1 which then bind and inhibits RAG GTPase complexes required for mTORC1 activation (Chantranupong et al., 2015). The location of both AMPK, GCN2 and Sestrin2/GATOR2, as well as many other growth factors pathways, upstream of TOR indicates that TORC1 and 2 are likely the center points mediating growth and environmental signals.

Although much is known about the cytosolic sensing of nutrients, how do these sensors link to the genome and impact gene expression? Furthermore, does the activation/inactivation of different proteins from these pathways stimulate the same sets of genes or impact the genome using common mechanisms? Are there tissue specific targets that mediate differing responses dictating how a specific cell type will react to changes in nutrient availability? When considering interfering with these pathways, does disruption at different points result in different patterns of gene expression and are these equivalent to CR itself? We know that signaling complexes such as TORC1 interact with a variety of transcription factors, including Hypoxia Inducible Factor 1α (HIF1α) and Peroxisome Proliferator-Activated Receptor γ (PPARγ summarized by Laplante and Sabatini, 2013). We will discuss some of these factors and their impact on genome function following CR or in response to CR mimetics.

## What are the mechanisms driving changes in gene expression following CR?

CR restriction in yeast stimulates stress response transcription factors Msn2/4 and Gis1. Under normal conditions, these factors are inhibited by TOR and the yeast homolog of the S6 kinase, Sch9 (Vidan and Mitchell, 1997; Roosen et al., 2005; Swinnen et al., 2006). However, deletion of Msn2/4 and Gis1 only partially impact the CR stress response, indicating that other factors also mediate this reaction (Wei et al., 2008). Further, signaling to transcription factors is also indicated in *Sch9* deletion mutants which demonstrate a 10-fold increase in lifespan (Wei et al., 2008). CR induces the function of the pyrazinamidase/nicotinamidase 1 (PNC-1) enzyme which deaminates and depletes nicotinamide (Anderson et al., 2003). Over expression of PNC-1 under normal nutrient conditions extends yeast lifespan by 70%. The induction of PNC-1 was not affected by deletion of the Msn2/4 transcription factors, indicating that these factors are dispensable for PNC-1 mediated lifespan extension under conditions of decreased glucose.

One of the major factors activated under conditions of nutrient sensing is the Sirtuin (SIRT) family of deacetylase proteins that are dependent on NAD+ which are high under conditions of nutrient deprivation (B'Chir et al., 2013). Although primarily known for their role as a histone deacetylase, SIRT proteins also target a large number of nuclear and cytosolic proteins. The extension of lifespan in yeast is linked to PNC-1 mediated deamination of nicotinamide leading to the activation of the Sir2 (yeast homolog of mammalian SIRT1) protein (Gallo et al., 2004). In mice, CR up-regulates the expression of SIRT1 (Cohen et al., 2004; Nemoto et al., 2004) while over expression of SIRT1 promotes phenotypes resembling that of CR (Bordone et al., 2007). Parallel to the increased NAD+ levels, SIRT1 is further stimulated directly by activated AMPK which deacetylates the tumor suppressor p53, inhibiting transactivation and p53-mediated gene expression (Giannakou and Partridge, 2004; Demidenko et al., 2009). SIRT1 function may be pivotal in defining how cells respond to stress induced by CR to promote longevity, whereas other stresses, such as DNA damage, lead to p53-mediated senescence or apoptosis. Somewhat paradoxically, sir2Δ yeast mutants exhibit increased resistance to oxidative stress and heat, indicating a pro-longevity phenotype (Fabrizio et al., 2005). These findings may indicate that the impact of Sir proteins may have divergent roles in chronological (total time) vs. replicated (number of cell divisions) lifespan (Longo, 2009).

SIRT1 activation via CR also results in decreased levels of inflammation by down regulation of genes encoding cytokines (Spaulding et al., 1997; Kalani et al., 2006; Meydani et al., 2014). In macrophages, SIRT1 activation results in deacetylation of the RelA/p65 component of nuclear factor-κB (NF-κB) complex (Stein and Matter, 2011). NF-κB is normally sequestered in the cytoplasm by its repressor Inhibitor of κB (IκB). Under conditions of stress, IκB is phosphorylated by the IK kinase (IKK) complex, leading to IκB dissociation from NF-κB. IκB is degraded and NF-κB translocates to the nucleus where it binds DNA and facilitates increased gene expression of pro-inflammatory genes. In addition, increased NF-κB function is observed in cells from older individuals (Kriete et al., 2008; Bektas et al., 2014). Acetylation at residue lysine 310 of RelA/p65 activates NF-κB, whereas removal of this acetyl group by SIRT1 inactivates the complex resulting in decreased expression of pro-inflammatory genes (Opalach et al., 2010). CR attenuated the age-mediated effects of NF-κB, resulting in more inhibitor IκB and decreased levels of RelA/p65 in a number of model organisms (Opalach et al., 2010). Furthermore, CR caused a reduction in the constitutive activation of p65 observed in CT-2A malignant mouse astrocytoma cells, inducing apoptosis (Mulrooney et al., 2011), thus supporting the hypothesis that CR is a viable treatment strategy for patients with aggressive brain cancers (Mukherjee et al., 2004).

The forkhead family transcription factor, FOXO3a, also known as DAF-16 in *C. elegans*, is a key regulator of the insulin receptor (IR)/insulin-like growth factor-I signaling pathway mediated extension of lifespan. CR stimulates the hyper-phosphorylation of FOXO3a leading to exclusion from the nucleus. This exclusion from the nucleus corresponded to an attenuation of Alzheimer's disease in Tg2576 mice (a specific model strain used for the study of Alzheimer's disease), indicating a link between CR, FOXO3a and age-related neurological pathologies (Qin et al., 2008). Other mammalian homologs of *Daf-16* in mice; however, show signs of increased expression during CR, indicating that these factors may be involved in promoting increased longevity in muscle cells (Furuyama et al., 2002). However, this study did not specify if these factors were prevented from becoming nuclear, indicating that there may be a cytosolic role for FOXOs, facilitating autophagy and extending lifespan. In addition, SIRT2 deacetylates FOXO3a in mice (Brunet et al., 2004; Daitoku et al., 2004; van der Horst et al., 2004), resulting in increased DNA binding of FOXO3a and expression of FOXO target genes, *p27-Kip1, manganese superoxide dismutase* (Kops et al., 2002), *catalase* (Furuyama et al., 2000; Nemoto and Finkel, 2002), and under conditions of severe stress, deacetylates and activates the pro-apoptotic protein Bim/BCL2L11 (Wang et al., 2012). In addition to these genes, FOXO3a also targets a variety of genes involved in several processes, including G1 arrest, G2 delay, DNA repair, ROS response, and glucose metabolism. Expression of SIRT1 is also dependent on the physical interaction of FOXO3a with p53 to promote binding within the SIRT1 promoter under conditions of decreased nutrient availability (Nemoto et al., 2004). In a separate experiment, knockout of FOXO3a in mice demonstrated that they no longer benefit from CR, indicating that this transcription factor is central in regulating genes that promote increased health and lifespan (Shimokawa et al., 2015), possibly mediating the interaction and functions of SIRT1 and p53. These observations strongly link SIRT1, p53, and FOXO3a in mediating a stress response in cells that favors a decrease in cell growth and proliferation without resulting in cell death.

Members of the PPAR nuclear receptor family PPARα, PPARβ/δ, and PPARγ are involved with regulating insulin sensitivity, adipogenesis, lipid metabolism, and blood pressure (Brown and Plutzky, 2007). Numerous polymorphisms have been identified in the PPAR genes that are directly related to type II diabetes and metabolic syndrome, indicating the pivotal role of these receptors in regulating gene expression in response to diet. PPARγ directly interacts with SITR1 under normal conditions. Diets which promote the formation of advanced glycation end products (AGEs) in diabetic patients and older individuals causes a decrease in both of these proteins (Singh et al., 2014). This decrease in PPARγ and SIRT1 further corresponds to an increase in inflammation. Restricting the dietary intake of AGEs in combination with CR re-established SIRT1 and PPARγ expression, decreasing the inflammatory response (Uribarri et al., 2015). CR as a treatment for disease represents a specific challenge when considering PPAR members. Although CR reduces the activity of PPARs through decreased activation, this leads to a decrease in the expression of genes regulating fatty acid metabolism. This results in the build-up of fatty acids in the liver causing steatosis/fatty liver disease. Paradoxically, Oshida and colleagues report that triglycerides directly activate PPARα during fasting or CR, both leading to cell proliferation and inhibition of inflammation (Oshida et al., 2015), demonstrating that both indirect and direct mechanisms control PPAR function.

CR decreases the expression of HIF-1α–mediated genes (Chen et al., 2009). HIF-1α activity is associated with a variety of other physiological stimuli such as heat acclimation, acidosis, nitric oxide exposure, inflammation and oxidative stress (Reviewed by Kang et al., 2005). In addition, HIF-1α activity is increased in aged rats, up-regulating genes such as *heme oxygenase-1 (HO-1), vascular endothelial growth factor (VEGF), erythropoietin (EPO)*, and *inducible nitric oxide synthase* (*iNOS*; Kang et al., 2005), with CR demonstrated to decrease the expression of these genes. CR may facilitate this process through the inhibition of mTOR signaling and decreased protein translation. Several studies have demonstrated that HIF-1α translation is dependent on mTOR activity (Bernardi et al., 2006; Hui et al., 2006). Repression of HIF-1α, therefore, leads to decreased levels of several genes including those regulating cell cycle progression, angiogenesis, and glucose metabolism (reviewed in Hong et al., 2004). In addition, SIRT1 (which is up-regulated under conditions of CR) deacetylates HIF1α preventing recruitment of the acetyl transferase p300 and further inactivation of HIF-1α target genes (Lim J. H., et al., 2010). HIF-1α also interacts with the redox factor REF-1, which is also involved with NF-κB signaling, indicating that both HIF-1α and NF-κB may respond in parallel under nutrient-rich conditions. Therefore, the repression of both the NF-κB and HIF-1α responses by CR may be analogous reactions to prevent inflammatory or stress responses that could potentially lead to carcinogenesis.

## Genome wide impact of CR on gene expression/gene pathways

A large number of gene expression studies have been performed in order to determine the impact of CR on genome function. CR is well known to elicit a change in cell behavior marked by a decrease in cell proliferation and shift to cellular maintenance and repair. Changes in phenotype are accompanied by changes in gene expression; therefore, what impact does CR have on gene expression from across the genome? CR of yeast caused 646 genes to significantly change expression leading to lifespan extension (Choi et al., 2013). These genes were identified to be positively correlated with transcriptional regulation, ribosomal processing and genome stability and negatively correlated with pathways involved with metabolism or cell cycle progression. Analysis of these genes indicated that the transcription factors Azoospermia Factor 1 (AZF1), Heatshock Factor 1 (HSF1), and X-box binding protein (XBP1) were involved with mediating lifespan extension under conditions of CR due to decreased glucose levels (Choi et al., 2013). These factors are involved with regulating stress response, indicating that CR may function as a cellular stress which promotes hormesis in yeast.

Whitaker and colleagues demonstrate that the timing of the gene expression analysis is critical in determining which genes are central in mediating increased longevity in response to CR (Whitaker et al., 2014). It was suggested that a small number of genes will rapidly be induced while secondary effects from initial changes would be observed at later time points. In this study, 853 genes were identified that changed expression over a 40 day period in *D. melanogaster*. Genes that changed expression demonstrated enrichment in pathways associated with folate biosynthesis, ubiquinone/terpenoid-quinone biosynthesis, and oxidative phosphorylation (Whitaker et al., 2014). Of these genes, very few were in common with starvation. Those that were common appeared to change expression in the opposite direction indicating that the response to CR is divergent from extreme nutrient deprivation (Whitaker et al., 2014). In support of this, starvation of *D. melanogaster* larvae exhibited differential activation of specific genes (Zinke et al., 2002) indicating that different levels of nutrients or duration may also impact expression profiles.

In an another meta-analyses, Swindell and colleagues identified more than 10,000 differentially expressed genes from over 40 independent datasets (Swindell, 2009). A meta-analyses of gene expression profiles in mammalian systems demonstrated that CR resulted in gene expression changes involved with a number of biological pathways, including growth hormone signaling, lipid metabolism, immune response, retinol metabolism, copper ion detoxification, and circadian rhythms (Whitaker et al., 2014). The large number of genes observed to have changed expression is likely due to many factors, including the use of expression data from different species, tissues and time points as well as also identifying secondary effects. It is more likely that in response to CR a few genes will have altered expression which leads to changes in cellular function and other secondary effects.

The identification of CR influencing circadian rhythm pathways (Swindell, 2009) and genes related to circadian rhythms is intriguing, demonstrating that diet can also affect sleep patterns, further impacting health. H3K4me3 deposited by the Mixed-lineage Leukemia 1 (MLL1) methyltransferase regulates the binding of the core transcription factors, circadian locomotor output cycles kaput (CLOCK) and BMAL1, controlling the cyclic expression of clock-controlled genes (CCGs). Indeed, the link between diet and circadian rhythms is likely mediated through SIRT1 function, with NAD+ levels influencing the epigenetic modification and expression CCGs therefore impacting biological rhythms (Aguilar-Arnal et al., 2015). Furthermore, AMPK phosphorylates the cryptochrome protein, resulting in the inhibition of CLOCK-mediated CCG expression (Masri and Sassone-Corsi, 2013). This provides further evidence for levels of cellular energy being essential in regulating health promoting biological processes such as circadian rhythms.

Contrary to the finding of Swindell and colleagues, another meta-analysis of microarray datasets from mouse, rats and pigs measuring gene expression changes as a result of CR demonstrated that only a handful of genes (101 up-regulated and 73 down-regulated) across multiple experiments. The Gene Ontology (GO) terms for these genes demonstrated enrichment in processes related to lipid metabolism and Acetyl-CoA reductase activity (Plank et al., 2012). In addition, terms for processes related to circadian rhythms were also identified in over expressed genes while down regulated genes showed enrichment for GO terms related to steroid biosynthesis, reduced transcription of genes involved with sterol biosynthesis and innate immune-response. However, the low number of genes identified over meta-analyses may reflect the heterogeneity created by using cross species comparisons and more candidates involved with CR may have been overlooked. Microarray analysis of multiple mouse tissues also indicated that there are tissue specific changes in gene expression related to CR (Park and Prolla, 2005). In an additional study of gene expression of muscle from male mice (Weindruch et al., 2001), microarray analysis demonstrated that only 58 genes were up-regulated and 55 genes were down-regulated. Of the 58 up-regulated genes, 16% were related to stress response and coincided with an increase in ROS produced in older mice. Many of these genes were either completely or partially repressed in CR mice fed 76% of the calories of mice on control diets. CR itself induced the expression of 51 genes, many of which were related to energy metabolism. Furthermore, CR increased the expression of genes involved with detoxification, which are hypothesized to decrease normally with age (Fu and Klaassen, 2014). An examination of 98 xenobiotic processing genes not only demonstrated an increase in expression of these genes, but also that CR favored the expression of female pre-dominantly expressed genes over pre-dominantly male expressed detoxification genes in the livers of male mice. These findings indicate that the CR may be inducing a shift toward maintenance and repair and re-establishing the expression of genes involved with detoxification, supporting the hormesis model.

Despite this plethora of publications regarding the impact of CR on genome function, there appears to be little consensus as to the genes that are responsible for promoting increased health and lifespan. This may be due to the complexity of the responses and differing mechanisms not only between cell types within the same organisms but also differing response between systems. Furthermore, the observation by Whitaker and colleagues indicating that the timing of analysis will be pivotal (Whitaker et al., 2014) highlighting the challenges underling the identification of genes controlling CR mediated impacts on health and lifespan. We will also note at this point that there is still controversy surrounding the impact of CR on longevity. For example, Harper et al. (2006) suggest that CR has no significant impact on mice outside of the laboratory setting. One explanation for this is that the CR regimes imposed by laboratories is too strict when compared to wild mice. Furthermore, wild mice also have more complex genetic backgrounds and diversity in their populations. To further complicate these issues, Mattison et al. (2012) indicated no impact on the survival of young or old rhesus monkeys exposed to CR. Regardless, both mice and rhesus monkeys did demonstrate an increase in healthspan and fewer age-associated pathologies in response to CR, arguing that although a statistical significance in lifespan is debatable, the evidence supporting a positive impact on health is strong. The complexities of these analyses may indicate that each cell/tissue type may have different genes that are active or inactivated as a result of CR.

## Epigenetic changes in response to CR

Critical to this discussion is the subject of epigenetics. Although the primary sequence of the genome is of obvious importance, with mutations in critical genes either promoting or shortening health and lifespan, it is not the only consideration when contemplating the impact of CR or CR mimetics. During the aging process our epigenome, the collection of epigenetic marks including histone modifications and CpG island methylation, is altered. For example, there is a genome-wide loss of histone 3 lysine 27 trimethylation (H3K27me3) and CpG island methylation resulting in the reactivation of repressed genes. This represents a loss of gene regulation, thus promoting cancer development (Issa et al., 1994, 2001; Knapowski et al., 2002). In prematurely aging HGPS cells, there is a distinct lack of heterochromatin with genome-wide loss of gene regulation (Kubben et al., 2012). Paradoxically, there are some CpG islands near promoters that become hypermethylated during aging (Singhal et al., 1987; Johnson et al., 2012), which results in the repression of nearby genes. Although the accumulation of mutations over time will impact genome function, another major driver of cellular aging is the loss of repressive epigenetic marks. These changes in epigenetic marks lead to changes in gene expression profiles, therefore, it is important to consider how CR impacts methylation and epigenetic marks across the genome.

Calories and nutrients from our diet are one of the most reliable, efficient and profound environmental factors leading to alteration in health and lifespan (Weindruch et al., 1986; Sinclair, 2005). CR is able to mediate specific changes in gene methylation profiles. For example, hypermethylation of the *c-myc* oncogene was observed in mice exposed to CR (Miyamura et al., 1993), which also lead to hypermethylation of the *ras* oncogene in rat pancreatic cells (Haas et al., 1993). These observations indicate that diet and cellular energy levels impact some of the most well characterized regulators of cancer and that this link is not a new phenomenon. Furthermore, the diets of our parents will play a large role in the profiles of our genomic function regardless of mutations within genes. A strong piece of evidence supporting the importance of epigenetics is the study from the Kim group; genome-wide sequencing of super-centenarians (110 years or older) demonstrated no significant enrichment for any single rare genetic variant to explain this longevity as compared to control genomes (Gierman et al., 2014). It is, therefore, likely that epigenetic modification to either histones or CpG islands due to environmental or maternal influences are responsible. In addition, CR prior to or during pregnancy can greatly impact the health and life span of offspring. CR of parents either prior to or during pregnancy has also shown to have benefits in the offspring, further supporting the role of epigenetics in regulating and increasing lifespan. Transgenerational epigenetic inheritance (the impact of environment on epigenetic status that is then passed to offspring) has been described in a diverse range of organisms ranging from plants to mammals. In *C. elegans*, knockdown of chromatin modifiers related to H3K4me3 in the F0 generation showed increased lifespan of offspring in the F3 generation, indicating a role for epigenetics in controlling inherited longevity (Greer et al., 2011). However, this does not appear to be the case with CR. In mice, CR prior to (1 month before pregnancy) or during pre-pregnancy leads to decreased health in offspring two generations later (Ponzio et al., 2012; Palou et al., 2015). CR of non-human primates during pregnancy also impacts the development of kidneys in the offspring, leading to decrease numbers of glomeruli (Nijland et al., 2007). This change in kidney development also corresponded to changes in gene expression from a number of different pathways, including the up-regulation of steroid metabolism and mTOR genes, and down-regulation of genes associated with oxidative phosphorylation, amino acid metabolism and cytokine-cytokine receptor interactions (Nijland et al., 2007). Therefore, despite not always having a positive impact, it is clear that CR, as well as other environmental factors experienced by the parents, has transgenerational epigenetic impact on the offspring influencing health and lifespan.

SIRT1 may also play a role in the establishment and maintenance of heterochromatin as a function of aging and CR. Endogenous SIRT1 can mediate the deacetylation of H4K16 and H3K9 leading to increased levels of H3K9me3, histone H1 recruitment, facilitating heterochromatin formation (Vaquero et al., 2004). This links SIRT1 deacetylase function not only with the regulation of specific factors and decreased gene expression but also to repression of specific loci and heterochromatin maintenance. This link is strengthened through the observation SIRT1 deacetylates and activates SUV39H1 methyltransferase (Vaquero et al., 2004, 2007) responsible for the deposition on methyl marks on H3K9 residues. This indicates that SIRT1 activity during CR is key in maintaining gene expression profiles through the deacetylation of genomic regions as well as the modulation of other proteins involved with chromatin structure.

What are some of the specific loci that are regulated by changes in epigenetic status in response to CR? In normal lung fibroblasts (WI-38) deprived of glucose, there is a marked change in epigenetic status with *p16-ink* promoter regions becoming less acetylated and hypermethylated. This change in acetylation and methylation causes *p16-ink* inactivation in leading to decreased cell proliferation (Li et al., 2010a). Contrary to this, the *human Telomerase* (*hTERT*) gene which encodes the main catalytic subunit of the telomerase enzyme, was activated becoming more acetylated and hypomethylated (Li et al., 2010a). These effects were opposite in immortalized cells, demonstrating a significant reduction in methylation with the *p16-ink* promoter and an increase in acetylation. The hTERT promoter became enriched for H3K9me3 and deacetylated in response to glucose withdrawal. The introduction of 5-aza-cytosine or trichsostatin A (TSA) reversed these effects, indicating that there is an epigenetic response of these promoters to glucose deprivation. Glucose deprivation (CR) promoted cell cycle arrest in normal fibroblasts while causing apoptosis in immortalized cells, demonstrating that glucose deprivation promotes maintenance and repair in normal cells while forcing cancerous cells to undergo apoptosis due to a lack of energy (Li et al., 2010a).

## Are compounds that have lifespan extension properties functioning through similar pathways to CR?

**Figure 3:**
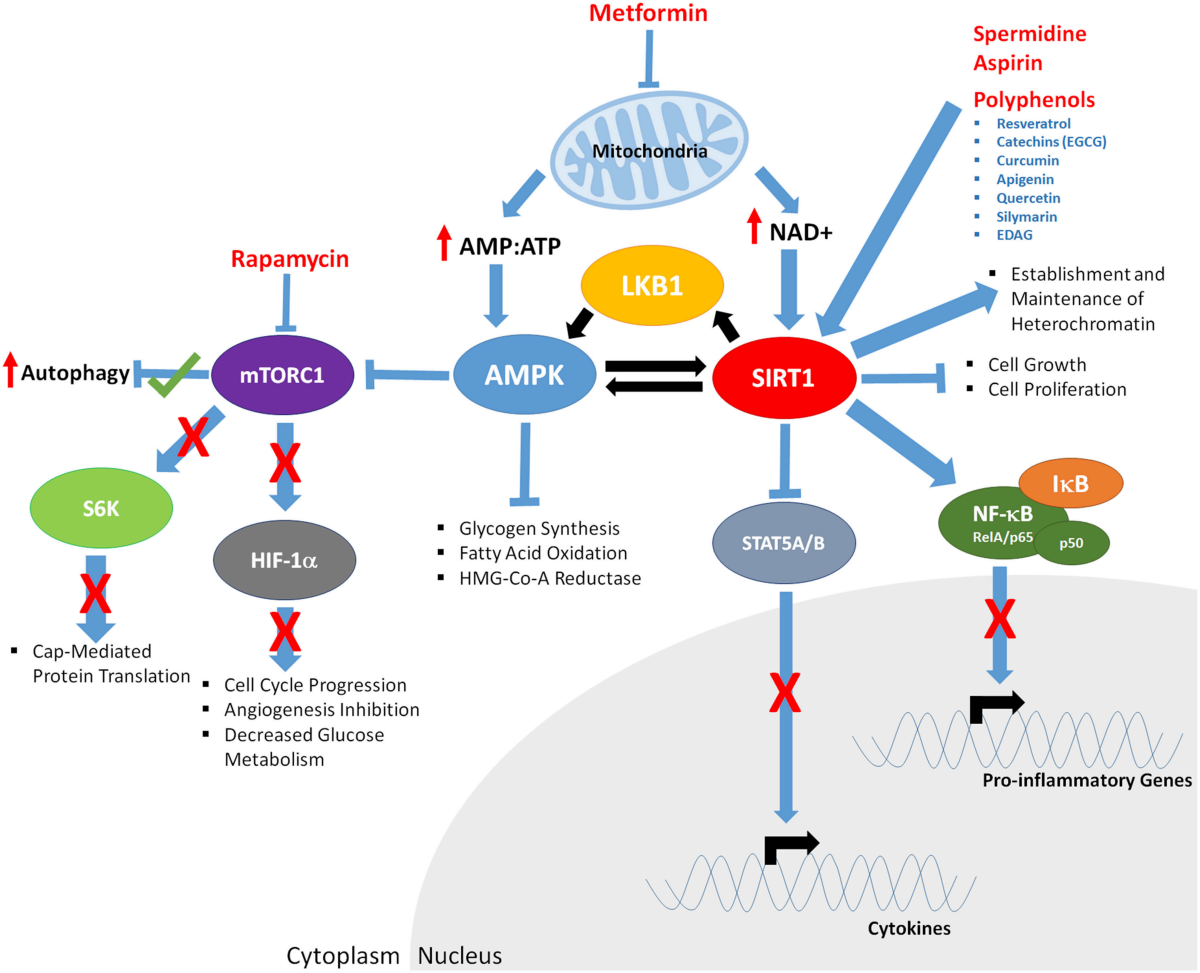
**The downstream effects of CR mimetics and nutraceuticals on key aging mediators AMPK and SIRT1**. Metformin exposure decreases mitochondrial function, increasing AMP:ATP ratios of and levels of NAD+. Increased AMP:ATP ratio leads to activation of AMPK. AMPK is phosphorylated/activated by LKB1 and SIRT1-mediated deacetylation, generating a feedback loop. AMPK inhibits glycogen synthesis, fatty acid oxidation, HMG-Co-A Reductase, and mTOR function (indirectly through phosphorylation of the TSC1/2 complex; not shown). Decreased mTORC1 function results in; increased autophagy, decreased S6K activity and cap mediated protein translation, inhibits HIF-1α, resulting in reduced cell cycle progression, angiogenesis, and glucose metabolism. Rapamycin mTORC1 kinase activity, potentially mirroring the effects of Metformin. Through decreased mitochondrial function, Metformin increases NAD+ levels, promoting SIRT1 activity, resulting in deacetylation of LKB1 and AMPK, and upregulated of their respective function. Polyphenols, Spermidine, and Aspirin increase SIRT1 activity, inducing deacetylation of the RelA/p65 component of NFκB, preventing degradation of IκB and sequestering NF-κB in the cytoplasm, inhibiting proinflammatory gene expression. Cytokine gene expression is repressed through SIRT1-mediated deacetylation and repression of STAT5A/B.

Cellular nutrient sensing is complex, with multiple proteins from several signaling cascades becoming active or repressed in response to changes in the levels of nutrients such as glucose, amino acids, hormones and cellular energy. Several pharmacological agents and naturally occurring nutricueticals can interact/inhibit/disrupt many of these proteins (Figure 3), disrupting the ability of cellular nutrient sensing. But do these compounds truly mimic CR?

## Rapamycin

In all model organisms tested thus far, caloric restriction has been shown to significantly down regulate mTOR function leading to increased autophagy and decreased protein translation (Blagosklonny, 2010). However, there are compounds that are hypothesized to mimic the effects of caloric restriction by disrupting a cells ability to sense nutrients in their environment. This is achieved as a result of mTOR inhibition, either directly or via activation/inhibition of factors up-stream or down-stream of mTOR complexes. The most well-known and characterized of these inhibitors is rapamycin. Rapamycin, a macrocyclic lactone-based compound, was first derived from the bacterium *Streptomyces hygroscopicus* found in the soil of Easter Island (Sehgal et al., 1975). Since its isolation, rapamycin has become extensively used as an immunosuppressant following organ transplantation (Dumont and Su, 1996). Rapamycin acts primarily through the TOR pathway; in mammals, rapamycin is bound between FKBP12 and the mTOR kinase subunits of mTORC1, causing functional inhibition of mTOR and the mTOR pathway (Dumont and Su, 1996). The downstream effects of this inhibition include a decrease in cap-mediated protein translation (Richter and Sonenberg, 2005) and an increase in autophagy (Jung et al., 2010), both associated with promoting health and lifespan. Rapamycin has been documented to ameliorate age related-disease phenotypes of numerous cell-based models (cancer; Shapira et al., 2006; Fang et al., 2011; Suzuki et al., 2011), cardiovascular disease (Das et al., 2014), the premature aging disease HGPS (Cao et al., 2011), neurodegenerative diseases (Santos et al., 2011) and extend lifespan in numerous model organisms including *S. cerevisiae* (Powers et al., 2006)*, D. melanogaster* (Bjedov et al., 2010), and *M. muscularis* (Miller et al., 2011, 2014; Zhang et al., 2014).

The accumulation of cells in senescence has been extensively linked to aging and the aging phenotype (Kong et al., 2011), with clearance of senescent cells documented to improve the symptoms of age-associated diseases (Baker et al., 2011). Mesangial cells (MC) became senescent in response to high glucose, increasing mTOR expression and decreasing SIRT1 expression (Zhang et al., 2012). It was demonstrated that rapamycin interfered with MC senescence coincident with increased SIRT1 expression (Zhang et al., 2012). In macrophages, inhibition of SIRT1 resulted in over expression of inflammation-related genes (*TNF*-α, *IL-6*) through NF-κB signaling activation. Rapamycin ameliorated SIRT1 inhibition, reducing NF-κB mediated inflammation (Takeda-Watanabe et al., 2012). The senescence associated secretory phenotype (SASP) involves secretion of pro-inflammatory cytokines from senescent cells into the local tissues environment, causing deleterious effects to surrounding cells and contributing to the aging process (Coppe et al., 2010). It is possible that SIRT1 is essential in regulating inflammation during the aging process, reducing inflammation-linked signaling in response to rapamycin treatment and promoting health and longevity. Similar evidence is present in response to CR (Spaulding et al., 1997; Kalani et al., 2006; Meydani et al., 2014).

Rapamycin has been extensively linked to cytokine expression and regulation across multiple cell lines. In healthy human foreskin fibroblasts [2DD], rapamycin caused up-regulation of numerous genes, including cytokine genes from the IL-6 signaling cascade, such as *IL-6, IL-8, IL-11*, and *leukemia inhibitory factor (LIF*; Gillespie et al., 2015). Analysis of Kyoto Encyclopedia of Genes and Genomes (KEGG) pathway terms identified a specific enrichment of cytokine-cytokine receptor interactions. Furthermore, transcription factor motif searches of up-regulated promoters and subsequent chromatin immuno-precipitation (ChIP) analyses demonstrated STAT5A/B as likely mediating rapamycin-induced changes in gene expression (Gillespie et al., 2015). Compounds that activate SIRT1 function suppress pSTAT5A/B signaling in response to *IL-2* and decrease mouse and human T-cell proliferation (Gardner et al., 2013). In orbital fibroblasts, rapamycin increased TNFα induced IL- 6 and IL-8 secretion by suppressing programmed cell death 4 (*PDCD4*) degradation (Lee et al., 2013), further increasing TNF, IL-6 and decreasing IL-10 levels in human macrophages. Contrary to this, in human oral keratinocytes (Su et al., 2015), rapamycin suppresses TLR3 and subsequent NF-κB signaling, inhibiting the production of interleukin-1 beta (*IL-1*β, *TNF*α, and *IFN*-β; but enhancing *IL-12p70* production (Zhao et al., 2010). Another study in senescent fibroblasts treated with rapamycin similarly demonstrated a decrease in *IL-6*/cytokine production, selectively suppressing translation of *IL-1A* (Laberge et al., 2015). These findings provide further evidence of SASP and the impact rapamycin may have on SASP to promote health and lifespan in cells. The conflicting impact of rapamycin on repressing or promoting gene expression/cytokine production may be complex and influenced by cell type and cells state (proliferative vs. senescent) in addition to the concentration and length of time in which cells have been exposed.

As with CR, rapamycin could have the opposite effect on cancer cells, resulting in cell death instead of promoting increased maintenance and repair. In oral squamous cell carcinoma (OSCC) Tca8113 and breast cancer cell lines, rapamycin down-regulated expression of S-phase kinase associated protein-2 (SKP2) and increased FOXO3a protein stability. When combined with cisplatin, rapamycin induced the up-regulation of feedback AKT activation-mediated FOXO3a phosphorylation leading to accumulation of p27 and Bim, increasing apoptosis (Shapira et al., 2006; Fang et al., 2011). The effects of rapamycin on these cancerous cells is parallel to the impact of CR inducing apoptosis previously identified. In healthy human cells, the apoptosis-promoting properties of FOXO are attenuated by SIRT1, further linking involvement of FOXO inhibition and SIRT1 deacetylase activity in moving cells away from cell death and toward survival (Giannakou and Partridge, 2004) in response to treatment with rapamycin and other longevity promoting compounds.

Reports have linked CR with mTOR signaling, resulting in increased expression of genes encoding proteins that protect against oxidative damage. It is possible that rapamycin treatment may induce similar effects. TOR function is associated with decreased transcription of stress response genes in *S. cerevisiae* via a mechanism involving Tap42 and the transcription factor Msn2 (Vidan and Mitchell, 1997; Roosen et al., 2005; Swinnen et al., 2006). Upon examination of rapamycin-treated mice spermatogonial stem cells (SSC), oxidative stress response genes were up-regulated (*SOD1, glutathione reductase*, δ*-aminolevulinate dehydratase*; Kofman et al., 2012). In older, wild-type mice, the expression of these genes was reduced. It is possible to conclude that oxidative stress genes are induced in response to rapamycin and may promote cellular longevity in SSC cells from mice (Kofman et al., 2012), possibly priming cells to manage alternative stresses, parallel to CR-mediated hormesis. Pre-treatment of lung carcinoma cells (A549) with rapamycin inhibited ROS and suppressed ROS-dependent apoptosis (Suzuki et al., 2011). In human corneal endothelial cells, rapamycin prevents cell-death as a result of oxidative injury via inhibition of ROS production (Shin et al., 2011) and although not directly reported, this was likely due to increased expression of genes encoding oxidative damage proteins. Although this links rapamycin to increasing health and lifespan, this link is divergent to that observed in CR. CR may improve health/lifespan by influencing protective enzymes, whilst rapamycin appears to inhibit ROS production and induce oxidative response genes.

Some controversy exists over the extent to which rapamycin impacts genome function in terms of gene expression. In healthy human fibroblasts treated with rapamycin for 5 days, 537 genes were identified by RNA sequencing with greater than five-fold change in expression (Gillespie et al., 2015); however, in the epididymal white adipose tissue of mice treated with rapamycin for 6 months, just 6 down-regulated genes were identified (Fok et al., 2014). This further supports the idea that rapamycin induces species/cell-type specific responses. It could also indicate that response to rapamycin is time specific, or even that the lack of genes significantly altered by long-term rapamycin treatment is the result of cells acclimatizing to long-term exposure. This is further supported through studies that document chronic exposure to rapamycin inducing inhibition of TORC2 under the same concentrations that only activate TORC1 under short-term exposure (Sarbassov et al., 2006).

Genome organization is tightly linked to genome function which not only includes interactions between specific genes/regulatory elements (Tolhuis et al., 2002; Schoenfelder et al., 2010; Zhou et al., 2014) but also the re-positioning of chromosome territories within the nuclear volume of cells. Chromosome territories rapidly re-locate in dermal fibroblasts in response to both quiescence induction via reduced serum and as cells become senescent (Mehta et al., 2010, 2011). The dynamic and rapid nature of this reorganization demonstrates the responsiveness of the genome to external stimuli. In the premature aging disease HGPS, treatment with rapamycin resulted in chromosomes re-positioning to a similar state to that of healthy cells, coincident with an improvement in disease phenotype (Cao et al., 2011). Furthermore, in healthy human fibroblasts, rapamycin induced repositioning of chromosomes 10 and 18 toward a more quiescent-like organization in parallel with an increase in population doubling times (Gillespie et al., 2015). The rapamycin-induced re-positioning of chromosomes was coincident with a significant change in gene expression. These findings link rapamycin-mediated inhibition of mTOR, chromosome territory positioning and gene expression profiles with the rate of cell growth. Furthermore, cells from HGPS patients lack heterochromatin and exhibited decreased DNA repair kinetics. This HGPS-associated loss of heterochromatin is parallel, although accelerated, to that occurring in normal aged cells. Cao and colleagues demonstrated that rapamycin restored heterochromatin domains in HGPS cells and DNA repair kinetics following the autophagy mediated degradation of the cytotoxic Progerin protein (Musich and Zou, 2011). This restoration of heterochromatin and DNA repair resulted in increased cellular lifespan. It is likely that changes in genome organization and the maintenance of heterochromatin will be observed under conditions of CR or as a result of CR mimetics that force changes in gene expression.

## Polyphenols

Polyphenols are portrayed as nutraceuticals in the media for their ability to act as free radical scavengers. This scavenging ability is due to the aromatic rings that are able to distribute charge over the molecule. It is theorized that ingested polyphenols are bioavailable in cells resulting in decreased metabolic ROS byproducts, reducing damage to molecules such as DNA. This scavenging ability is far from the only health promoting properties these molecules have. There are a number of naturally occurring polyphenols, including curcumin (Jobin et al., 1999), apigenin (Wang et al., 2014), quercetin (Endale et al., 2013), silymarin (Saliou et al., 1998), and (my favorite) 2α,5-epoxy-5,10-dihydroxy-6α-angeloyloxy-9β-(3-methylbutyloxy)-germacran-8α,12-olide (EDAG; Lee et al., 2011) that function to inhibit inflammation. Under conditions that induce inflammation, such as lipopolysaccharide treatment, NF-κB is released from its inhibitor IκB, translocates to the nucleus and promotes pro-inflammatory transcription. Collectively these polyphenolic compounds prevent the degradation of IκB, blocking NF-κB function and the inflammatory response. A comprehensive examination of the impact of several polyphenols on NF-κB signaling demonstrated, however, that differing conditions or cell types, such as intestinal Coca-2 cells, may have opposite effects (Romier et al., 2008).

## Resveratrol

Resveratrol (3,5,4′-trihydroxystilbene), is a well-known polyphenol commonly found in a variety of foods, including a number of berries, peanuts, grapes and red wine (Stervbo et al., 2007). In fact, the presence of resveratrol in red wine has been linked to the decreased levels of cardiovascular disease in the French population (Renaud and de Lorgeril, 1992). It has since been documented that resveratrol has beneficial effects on cancer, including the prevention of carcinogenesis in mice, and the reduction in proliferation in a number of cancer-based cell lines (prostate; Narayanan et al., 2003; Jones et al., 2005, ovarian; Yang et al., 2003, breast; Chin et al., 2014). Given that both cancer and cardiovascular disease are considered as age-related diseases, the consideration of resveratrol as a potential intervention for age-related diseases is realistic, with evidence already implicating resveratrol as beneficial across numerous areas of aging (e.g., cognitive aging in mice; Torres-Perez et al., 2015). Although it has been suggested that resveratrol has no impact on lifespan (Bass et al., 2007), several studies have reported lifespan extension across a number of model organisms, such as *N. furzeri* (a short-lived seasonal fish; Valenzano et al., 2006), *S. cerevisiae* (Howitz et al., 2003), *C. elegans*, and *D. melanogaster* (Wood et al., 2004). The mechanisms for this resveratrol associated lifespan extension are thought to be centered around activation of Sirtuin deacetylases (whether this activation be direct or indirect; Pacholec et al., 2010), which are linked to CR-mediated lifespan extension.

Resveratrol may also promote increased health by altering gene expression patterns. In the prostate cancer cell line, LNCaP, 1656 transcripts were identified as changing greater than two fold expression in response to resveratrol treatment, 37% up-regulated and 63% down-regulated (Jones et al., 2005). This change in transcript profile was coincident with a decrease in cell proliferation. Multiple labs have demonstrated that resveratrol impacts both the androgen (Narayanan et al., 2003; Jones et al., 2005; Wang et al., 2008) and estrogen (Gehm et al., 1997; Wang et al., 2008) receptor pathways. Androgens mediate development and physiological responses and have been associated with cellular functions such as cell cycle regulation, transcription, cell proliferation and differentiation, including down-regulation of *prostate specific antigen* (*PSA*; Jones et al., 2005; Wang et al., 2008) and *AR* and the AR co-activator *ARA* (Narayanan et al., 2003; Jones et al., 2005). Resveratrol may be altering expression of genes via inhibition of androgen and estrogen receptor-dependent signaling pathways (Wang et al., 2008). Resveratrol, via activation of FOXO transcription factors, induces growth arrest and apoptosis in LNCaP, with *in vitro* experiments inducing decreased tumor apoptosis and increased tumor angiogenesis despite initially delaying tumor growth (Wang et al., 2008; Chen et al., 2010). These observations, as well as others, demonstrate that resveratrol induces gene expression profiles that promote cell death in a number of cancer cell lines (Hsieh et al., 1999; Young et al., 2005; Chin et al., 2014). SIRT1 has been identified to deacetylate both the androgen (Fu et al., 2006) and estrogen (Yao et al., 2010) receptors in addition to FOXO proteins [notably resveratrol treatment induces expression of FOXO transcription factors, with SIRT1/FOXO demonstrated to be responsible for increased endothelial NO synthase (eNOS) which increases nitric oxide (NO) production and is attributed to improving cardiovascular health; Xia et al., 2013]. Given the proposed impact of SIRT1 activation, resveratrol could promote cell death of cancers by interfering with the expression of androgen and estrogen mediated genes as well as activating pro-apoptotic genes (such as BIM; Chen et al., 2010) through FOXO-mediated DNA binding.

In cancerous cell lines, the cytoprotective genes *NQ01* (Yang et al., 2003; Jones et al., 2005), and *PRDX1*, as well as *MGST1* and *GSTA2* were induced by resveratrol treatment (Jones et al., 2005). These genes are transcribed by NRF2, a member of the NRF transcription factor family. SIRT1 deacetylates NRF family members, therefore, it could be that the induction of these genes in response to resveratrol is the result of SIRT1 activation of NRF2. Furthermore, *JUNB, HSP40, SERP1*, and *STCH* were up-regulated in response to resveratrol, indicating cellular stress, with resveratrol inducing cell cycle arrest and apoptosis at higher doses (Jones et al., 2005). Low levels of resveratrol may prime cells to deal with DNA-damage causing agents; however, at higher levels become a stress itself.

Resveratrol may also mirror the function of CR and promote health and longevity at the genetic level by interfering with pro-inflammatory signaling pathways. In healthy cells and tissues, resveratrol is able to block TNF-induced activation of the nuclear transcription factor NF-κB, which regulates genes involved in inflammation, cytoprotection and carcinogenesis (Busch et al., 2012). This is likely through the activation of SIRT1 mediated deacetylation of the p65/RelA subunit of NF-κB. In human tenocytes, resveratrol suppressed IL-1b induced activation of NF-κB and PI3K, inhibiting genes involved in inflammation and apoptosis (Busch et al., 2012). Resveratrol further inhibited IL-1b induced NF-κB and PI3K activation through inhibition of IKK, IκBa phosphorylation and inhibition of nuclear translocation of NF-κB, implicating PI3K signaling as involved in the downstream impact of resveratrol (Busch et al., 2012; Ren et al., 2013). Resveratrol, as with other mimetics of CR, has been documented to have varied effects between healthy and cancer cell lines. In myeloid cells (U-937), Jurkat and epithelial cells (Hela and H4) resveratrol suppressed TNF-induced phosphorylation and nuclear translocation of p65/RelA. Also suppressed by resveratrol are AP-1,TNF-induced activation of mitogen-activation protein kinase kinase (MAPKK), TNF-induced cytotoxicity and caspase activation (Manna et al., 2000). Resveratrol activated p53 and influenced gene expression in LnCaP cells, impacting 34 transcripts being either up- or down-regulated. Many of the transcripts were involved with apoptosis [including *programmed cell death factor 2* (PDCD-2)*, p300, Apaf-1, CPP32, PIG 7, PIG8, BAK protein*, and *p57(Kip2)*] (Narayanan et al., 2003), with resveratrol further impacting gene expression (Le Corre et al., 2006), decreasing proliferation (Delmas et al., 2000), and inducing apoptosis (Niles et al., 2003) in a number of human cancers. This change in gene expression was coincident with decreased activation of NF-κB (Narayanan et al., 2003). Genes involved in DNA damage, cell cycle and oxidative stress were were also noted to be down regulated (Narayanan et al., 2003). These observations demonstrate that resveratrol is able to mimic CR by SIRT-mediated deacetylation of pro-inflammatory complexes such as NF-κB in normal cells while possibly having the opposite pro-apoptotic effect in cancer lines.

## Catechins

In Japan, subpopulations are long-lived; this may be due to the ingestion of large quantities of green tea enriched for in polyphenolic compounds. One of the most well studied of these polyphenolic compounds is (-)-epigallocatechin-3-gallate (EGCG). Like other polyphenols, it has free radical scavenging capabilities and inhibits NF-κB mediated inflammatory responses in a variety of systems (Akhtar and Haqqi, 2011; Han et al., 2012; Jiang et al., 2012), undoubtedly a major factor in this observed longevity. However, EGCG has other ascribed impacts that may contribute to increased longevity. EGCG reduces levels of adipogenesis-related transcriptional factors, such as C/EBPβ and PPAR, of differentiating 3T3–L1 preadipocytes (Kao et al., 2006). EGCG treatment of breast cancer cells can reactivate estrogen receptor (ER)-α through a reversal of epigenetic silencing, leading to apoptosis (Li et al., 2010b). In addition EGCG also directly and indirectly inhibits DNA methyl transferases (DNMTs) causing the loss of methylation from the *p16*^*ink*^ promoter, retinoic acid receptor β (RARβ), and the DNA mismatch repair gene human mutL homolog 1 (hMLH1). EGCG also promotes the repression of the gene encoding for a subunit of the human telomerase enzyme, hTERT, resulting in cancer cell senescence (Berletch et al., 2008), similar to suppression induced by glucose restriction of lung fibroblasts (Suzuki et al., 2011). A comprehensive review of EGCG functions and modes is given by Singh and colleagues describing the impact of this compound on cell cycle inhibition, apoptosis and growth factor signaling (Singh B. N. et al., 2011; Singh H. et al., 2011).

Unlike rapamycin or resveratrol, polyphenols in general may not promote longevity *per se*, but have anti-cancer properties. For example, genistein also stimulated the loss of *hTERT* expression by increasing H3K9me3 and decreasing H3K4me2 within the promoter region, preventing E2F-1-mediated transcriptional activation (Li et al., 2009), leading to decreased cancer cell proliferation. In addition, many of the actions of EGCG are related to promoting apoptosis or senescence of cancer cells through changes in epigenetic status (Berletch et al., 2008; Singh H. et al., 2011). Although these compounds are exciting potential chemotherapeutic agents, they may not be directly acting as anti-aging compounds.

## Metformin

Metformin, a compound derived from the French Lilac (*G. officinalis*) and chemically known as N,N-Dimethylimidodicarbonimidic diamide, is a guanidine-based hypoglycemic agent commonly used in treating patients with type II diabetes (T2D; Witters, 2001) by inhibiting hepatic gluconeogenesis and decreasing insulin levels (Hundal et al., 2000; Zhou et al., 2001). Despite being such a widely used compound, the specific mechanisms by which metformin act at the molecular/cellular level remain unconfirmed. Regardless, evidence does indicate that metformin functions, at least in part, via an AMPK-dependent pathway (Zhou et al., 2001). It is unlikely that metformin directly binds to either AMPK or its activator LKB1 as the drug does not affect phosphorylation of AMPK by LKB1 in a cell-free assay (Hardie, 2006). Metformin likely influences AMPK levels by modulating ATP production by mitochondria. Evidence to support this hypothesis indicates that metformin induces mild and specific inhibition of the mitochondrial respiratory-chain complex I (El-Mir et al., 2000; Owen et al., 2000). Metformin has further been suggested to act through a number of AMPK-independent pathways such as via DNA-damage inducible transcript 4 (DDIT-4; Ben Sahra et al., 2011). Although the positive health benefits in regards to T2D and potentially health and lifespan are well documented, how this is facilitated through numerous pathways and secondary effects is unclear. This metformin-mediated disruption of mitochondrial function may parallel CR through increased AMP:ATP ratios leading to AMPK activation (Canto and Auwerx, 2011) and mTOR inhibition.

Recent evidence has highlighted metformin as both a potential anti-cancer agent and as a promising target in promoting increased health and lifespan. Numerous studies report increased apoptosis and decreased proliferation of various cancer cell lines (e.g., lung; Ashinuma et al., 2012, retinoblastoma; Brodowska et al., 2014, and oesophageal squamous cell carcinoma; Feng et al., 2014) in response to metformin, and T2D patients treated with metformin have a lower incidence of cancer compared to control groups (Evans et al., 2005). This is promising given the extensive links between cancer and aging; however, healthy model organisms treated with metformin have shown varied responses, with lifespan extension occurring in *C. elegans* (Onken and Driscoll, 2010; Cabreiro et al., 2013) and *M. musculus* (Martin-Montalvo et al., 2013), whilst *D. melanogaster* (Slack et al., 2012) and *R. norvegicus* (Smith et al., 2010) demonstrated no change.

What impact does metformin have on genome function to promote health? Under conditions of excess nutrients AMPK and SIRT1 are down regulated. Metformin results in the activation of both these proteins (Nelson et al., 2012; Arunachalam et al., 2014) similar to CR (Cohen et al., 2004; Canto and Auwerx, 2011). The metformin-mediated activation of AMPK and SIRT1 deacetylation of p53 decreases its function in the human hepatic carcinoma cell line, HepG2 (Nelson et al., 2012). In addition, metformin appears to reduce levels of oxidative stress, impeding p53 activation. It also decreased a trigger for p53 accumulation, cytosolic oxidative stress and increased deacetylation of p53 at a SIRT1 targeted site (Nelson et al., 2012). Furthermore, mouse microvascular endothelial cells (MMEC) that had been exposed to high glucose were treated with metformin. This treatment up-regulated SIRT1 expression which is normally repressed by high glucose, modulating downstream targets of SIRT1 (*FoxO1* and p53/p21) and protecting endothelial cells from entering premature senescence (Arunachalam et al., 2014). In blood mononuclear cells (MNC) of patients with carotid artery atherosclerosis, metformin ameliorates the pro-inflammatory response decreasing *IL-6, TNF*-α mRNA levels and attenuated NF-κB DNA binding activity (Xu et al., 2015). Metformin did not alter p65/RelA protein levels but resulted in decreased acetylation, likely through SIRT1-mediated deacetylation (Xu et al., 2015). Additionally, *Saa1* and *Saa2* genes, associated with the inflammatory response, are down-regulated in the muscle and liver of metformin-treated mice. A decrease in inflammatory markers (attenuated expression of the *NF*-κ*B* gene, resulted in decreased NF-κB and JNK) was also observed (Martin-Montalvo et al., 2013). This is coincident with a significant up-regulation of *cytokine-inducible SH2-containing protein* (*CISH*), a negative regulator of cytokine signaling (Martin-Montalvo et al., 2013). Metformin has also been associated with the induction of stress-response and antioxidant-linked proteins, including SOD2, TrxR1, NQ01 and NQ02 in mice livers (Martin-Montalvo et al., 2013). Furthermore, metformin treatment decreased production of IL-1β, increased induction of anti-inflammatory IL-10 and inhibited ROS in macrophages (Algire et al., 2012; Kelly et al., 2015). Contradictory to CR, metformin resulted in increased levels of ROS which up-regulated the expression of the UCP2 transcripts in epididymal white adipose tissue of mice or in 3T3-L1 adipocytes (Anedda et al., 2008). Taken together, these data indicate that metformin may inhibit multiple pro-inflammatory pathways, through activation of SIRT1 and the repression of NF-κB in addition to the up-regulation of *CISH*, as part of the mechanism influencing health and longevity. As with rapamycin and CR, however, different systems and cell types may result in differing responses.

The expression of the Selenoprotein P (SeP) encoding *SEPP1* gene leads to increased potential in developing T2D (Takayama et al., 2014). Promoter analysis and subsequent reporter assays of this gene demonstrates binding sites for FOXO3a to facilitate the expression of this gene. Metformin treatment disrupted FOXO mediated expression of this gene through direct phosphorylation by activated AMPK (Takayama et al., 2014). *Daf-16* knockouts in *C. elegans* leads to increased lifespan by disruption of the insulin-like pathway (Greer and Brunet, 2005). CR results in FOXO3a hyperphosphorylation by AMPK and its exclusion from the nucleus. Metformin-mediated activation of AMPK also leads to hyperphosphorylation of FOXO3a, nuclear exclusion and up-regulation of mitochondrial gene expression (Greer and Brunet, 2005). However, knockout of *daf-16/FOXO* still resulted in an increase in median lifespan, demonstrating that this transcription factor is dispensable for metformin mediated lifespan extension (Onken and Driscoll, 2010). Therefore, metformin may be acting through a CR conferred pathway. In *eat-2* knock-outs (which impair the ability of worms to feed) treated with metformin, no health or lifespan benefits were observed and detrimental effects similar to those of extreme CR were documented. Regardless, the extension of lifespan requires functional AMPK (Onken and Driscoll, 2010) indicating that this is a critical molecule in metformin mediated lifespan extension.

Knockout and mutational analysis of the *C. elegans* homolog of AMPK, *aak-2*, demonstrates that this protein is essential for metformin-associated lifespan extension (Onken and Driscoll, 2010). Furthermore, the protein threonine kinase, LKB1, directly phosphorylates and activates AMPK in response to metformin. In C. elegans, PAR-4 (the LKB1 homolog) mutants treated with metformin conferred no health or lifespan benefits (Onken and Driscoll, 2010) demonstrating the importance of this kinase in mediating AMPK function. In the absence of this LKB1 activity, metformin treatment resulted in decreased lifespan. LKB1 inactivation has been suggested to be regulated by SIRT1, influencing its cytosolic localization, association with the LKB1 activator STE20-related adaptor (STRAD), kinase activity and its ability to activate AMPK (Lan et al., 2008). This links the interplay between AMPK, LKB1, and SIRT1 as key mediators of longevity across model organisms.

To ascertain what impact metformin treatment is having genome-wide, transcript profiles from LoVo colon cancer cells were assessed by microarray analysis (He et al., 2015). At 8 h, 10 mM metformin resulted in 134 differentially expressed genes, whilst at 24 h 3061 genes altered expression. Concentration of metformin also demonstrated alternative impacts on health and lifespan with 0.1% (w/w) metformin in the diets of C57BL/6 increasing lifespan whilst 1% (w/w) metformin decreased lifespan (Martin-Montalvo et al., 2013). In B6C3F1 mice, lifespan extension was also observed; however, the levels were less significant (Martin-Montalvo et al., 2013). Microarray analyses of the muscle and liver cells of metformin-fed mice revealed a transcriptome profile shifting toward that, but not identical to, caloric restriction by 30 weeks. Although *CISH* was consistently one of the most up-regulated genes in both tissues (Martin-Montalvo et al., 2013), other genes were up-regulated in one but not the other (e.g., *SOCS2* up-regulated in liver, *DDIT4* in muscle). Therefore, the effects of metformin are not only strain/tissue-, dose- and time-dependent, but may also not directly mimic CR.

Gene ontology (GO) term enrichment of LoVo cells treated with metformin revealed up-regulated genes enriched for processes such as RNA processing and regulation of cellular protein metabolic process whilst down-regulated genes demonstrated enrichment for various cell cycle processes at 8 h. At 24 h, up-regulated genes were enriched for in pathways of cell activity (e.g., protein modification, induction of apoptosis and response to reactive oxygen species; He et al., 2015). Microarray analysis of mouse liver tissues following 0.1% (w/w) metformin treatment revealed down-regulation of genes associated with autophagy, and apoptosis in mice (Martin-Montalvo et al., 2013). Although this is an opposite impact to that observed in LoVo cells, it is logical and in agreement with the proposal that metformin induced apoptosis in cancer cell lines, whilst in healthy tissues, promotes cell survival, likely via SIRT1/FOXO axis.

## Spermidine

In general, polyamines are polycationic molecules that interact with negatively charged polymers such as DNA, RNA and proteins and can be found in a large number of eukaryotic and prokaryotic organisms (reviewed in Minois, 2014). Polyamines decrease with aging in several mouse tissues (Nishimura et al., 2006) and in the aging human basal ganglia (Vivo et al., 2001). Although polyamines, including spermidine, are naturally produced within cells, ingestion of polyamines from the diet can impact gene expression and chromatin organization associated with increased healthspan and longevity (Minois et al., 2011). The specific mechanisms by which these molecules facilitate changes in gene expression are indirect or have not been elucidated. Like CR, and other nutraceuticals that impact lifespan, spermidine reduces the expression of pro-inflammatory genes. This suppression of pro-inflammatory genes is hypothesized to be the result of sequestered NF-κB subunit p65 in the cytoplasm, preventing the activation of target genes (Choi and Park, 2012). In 3T3-L1 cells, spermidine has been shown to directly interact with the acidic nuclear phosphoprotein 32 (ANP32; Hyvonen et al., 2013). Upon spermidine interaction, ANP32 releases the RNA binding protein HuR which then binds and facilitates the translation of C/EBP-b. This increase in C/EBP-b speculated to further drive the expression of genes encoding the transcription factors PPAR-γ and SREBP-1c leading to adipocyte differentiation (Hyvonen et al., 2013). Furthermore, transcriptome profiling of cells depleted of polyamines demonstrated increased expression of cell cycle control proteins p21, Mdm2, and Gadd45 as well as reduced expression of cyclin D1 (Landau et al., 2012). However, it is unclear if these changes were secondary effect of polyamine depletion activating the unfolded protein stress response. These stress responses and resumption of cell growth were quickly reversed upon the addition of spermidine.

Spermidine also appears to impact global acetylation levels of histones. In yeast, all histone H3 lysine residues studied showed decreased levels of acetylation following spermidine treatment in addition to decreased levels of ROS. This hypoacetylation is believed to be due to spermidine mediated inhibition of histone acetyl transferases (HATs) leading to increased expression of the autophagy-related *ATG* genes (Eisenberg et al., 2009). Furthermore, keratinocytes from mice over-expressing enzyme promoting spermidine production demonstrated an overall reduction in acetylation levels indicating a further impact on gene expression. Although this decrease in acetylation levels as well as the inhibition of NF-κB is reminiscent of SIRT1 activation, recent research has proposed that spermidine and other SIRT1 activators (such as resveratrol) function via differing pathways, but both impacting global protein acetylation (Morselli et al., 2011). Spermidine also has a complex nature with regards to cancer cells. In some cancers polyamines synthesis is up-regulated resulting in an indirect increase p300-associated HAT activity, altering chromatin structure favoring neoplastic process (Hobbs et al., 2002).

## Aspirin

Aspirin, also known as acetylsalicylic acid (ASA), is a member of the non-steroidal anti-inflammatory (NSAID) drug family and is well known for its inhibitory effect on *COX-2* gene expression. *COX-2* encodes the cyclooxygenase 2 enzyme which functions to convert arachidonic acid to prostaglandins which further function in pain and inflammatory responses. Nuclear translocation of activated NF-κB promotes the transcription of target genes such as *Cox*-2, *iNOS, VCAM*-1, and *ICAM*-1. Low doses of ASA fed to old rats appeared to ameliorate the NF-κB-mediated pro-inflammatory response in kidneys by preventing IκB degradation, leading to cytoplasmic retention of NF-κB (Jung et al., 2006). In addition, ASA also appeared to inhibit the nuclear translocation of the complex containing thioredoxin (Trx) and redox factor-1 (Ref-1) which enable DNA binding of NF-κB. Investigation into other compounds closely related to ASA from the NSAID family have focused on the impact of these drugs on NF-κB function. Furthermore, treatment of breast cancer cells with the physiological achievable concentration of 100 μM ASA increased levels of p53 acetylation and increased the expression of p21^CIP^ (cell cycle arrest) and Bax (pro-apoptotic). ASA may further increase health and lifespan by inducing the expression of pro-apoptotic genes in cancer cells. For example, ASA has been documented to up regulate *calpain* expression in cervical carcinomas. Calpains are a class of non-lysosomal cysteine proteases involved with a number of cellular processes including glucose homeostasis. Furthermore, calpains exhibit cross talk with the pro-apoptotic protease caspase-3 (Lee et al., 2008). However, the response of cells/tissues to ASA may not be straight forward. Microarray analysis of HT29 colon cancer cells demonstrates significantly different transcriptome profiles when treated with 50 μM, 500 μM, or 5 mM of ASA (Hardwick et al., 2004). These observations indicate that ASA is potentially useful as a chemo-preventative agent for cancers and, therefore, involved in promoting health, but the dose of the compound may alter gene expression profiles leading to alternative, possibly undesired outcomes. In addition, risk-benefit research into the use of ASA indicates that patients with disease that make them prone to cancers, such as familial adenomatous polyposis or Lynch syndrome, are not protected (Alfonso et al., 2009). Other cancer types, such as adenomas, do show statistically significant alterations in response to ASA. These observations, indicate that ASA does promote changes in genome function leading to increased health and possibly the prevention of specific cancers; however, this role in specifically increasing longevity *per se* is unclear.

## Conclusions

It is clear that CR results in decreased energy and changes in cellular AMP:ATP and NAD:NADH ratios. Compounds that mimic CR do so by impacting cellular function resulting energy readouts or interfering with signaling down-stream cellular energy levels. The main proteins that appear central to mediating this response are AMPK and SIRT1 which regulate cycles of deacetylation and phosphorylation of a large number of cytosolic and nuclear proteins to control gene expression and cellular functions. Many of the CR mimetics of naturally occurring compounds identified either modulate SIRT1/AMPK function or, for example with rapamycin, target downstream signaling hubs to mediate potential health and lifespan effects. Of these targets NF-κB and the FOXO family of transcription factors, are pivotal in promoting decreased cell proliferation and increased maintenance in normal cells, while facilitating apoptosis and cell death in cancer cells. Furthermore, although all compounds appear to confer life and healthspan extending impacts across numerous cell types and model organisms via this SIRT1/AMPK interaction, the downstream impact on genome function (gene expression) is varied, across cell-type, organism-type, and compound-type in addition to variations in experimental details (such as exposure times, drug concentrations). This suggests that although mechanisms mediating health and lifespan in response to CR and these compounds are similar, the effects on gene expression mean that these compounds may not be direct mimetics of CR or of one another.

## Author contributions

ZG, JP, and CE constructed and wrote the manuscript. All authors contributed to the editing and final submission of the document.

## Funding

This work was supported by the NSERC (grant number RGPIN-2015-04930) Discovery Grant program.

### Conflict of interest statement

The authors declare that the research was conducted in the absence of any commercial or financial relationships that could be construed as a potential conflict of interest.
